# Genetic separation of southern and northern soybean breeding programs in North America and their associated allelic variation at four maturity loci

**DOI:** 10.1007/s11032-016-0611-7

**Published:** 2017-01-11

**Authors:** Goettel Wolfgang, Yong-qiang Charles An

**Affiliations:** US Department of Agriculture, Agricultural Research Service, Midwest Area, Plant Genetics Research Unit at Donald Danforth Plant Science Center, 975 N Warson Rd, St. Louis, MO 63132 USA

**Keywords:** Soybean, breeding history, Pedigree, Breeding, Network, *E* genes and maturity

## Abstract

North American soybean breeders have successfully developed a large number of elite cultivars with diverse maturity groups (MG) from a small number of ancestral landraces. To understand molecular and genetic basis underlying the large variation in their maturity and flowering times, we integrated pedigree and maturity data of 166 cultivars representing North American soybean breeding. Network analysis and visualization of their pedigree relationships revealed a clear separation of southern and northern soybean breeding programs, suggesting that little genetic exchange occurred between northern (MG 0–IV) and southern cultivars (MG V–VIII). We also analyzed the transcript sequence and expression levels of four major maturity genes (*E1* to *E4*) and revealed their allelic variants in 75 major ancestral landraces and milestone cultivars. We observed that *e1-as* was the predominant *e* mutant allele in northern genotypes, followed by *e2* and *e3*. There was no allelic variation at *E4*. Transcript accumulation of the *e2* mutant allele was significantly reduced, which might be caused by its premature stop codon triggering the nonsense-mediated mRNA decay pathway. The large DNA deletion generating the *e3* mutant allele also created a gene fusion transcript. The *e* alleles found in milestone cultivars were traced through pedigrees to their ancestral landraces and geographic origins. Our analysis revealed an approximate correlation between dysfunctional alleles and maturity groups for most of the 75 cultivars. However, single *e* mutant alleles and their combinations were not sufficient to fully explain their maturity diversity, suggesting that additional genes/alleles are likely involved in regulating maturity time.

**Electronic supplementary material:**

The online version of this article (doi:10.1007/s11032-016-0611-7) contains supplementary material, which is available to authorized users.

## ᅟ

Soybean (*Glycine max* (L.) Merrill) is a photoperiod-sensitive plant that flowers in response to shorter day length. Soybean cultivars have to acquire photoperiodic insensitivity to flower and produce seeds at higher latitudes (Xu et al. 2013). Soybean was domesticated from its wild relative *Glycine soja* in East Asia several thousand years ago. In contrast, soybean has a rather short history in North America. Soybean was only introduced to North America in the seventeenth century and was mostly grown as a forage crop until the 1920s. The first modern soybean cultivar developed by hybridization in North American breeding programs was released in 1939 (Bernard et al. 1988). The transition from selecting landraces to breeding cultivars resulted in a significant genetic improvement of soybean cultivars (Rincker et al. 2014). During soybean domestication and breeding, soybean cultivars with a wide range of flowering and maturity time were developed. Current soybean cultivars have been bred to grow in latitudes ranging from the equator to 50° N and higher (Tsubokura et al. 2013). In general, a given cultivar is developed for maximum yield potential within a specific latitude range. Cultivars are assigned to specific maturity groups ranging from 000 to X, which indicate their preferred latitudinal or geographic zones in North America from Southern Canada (000) to Mexico and the Caribbean Islands (X).

**Fig. 1:**
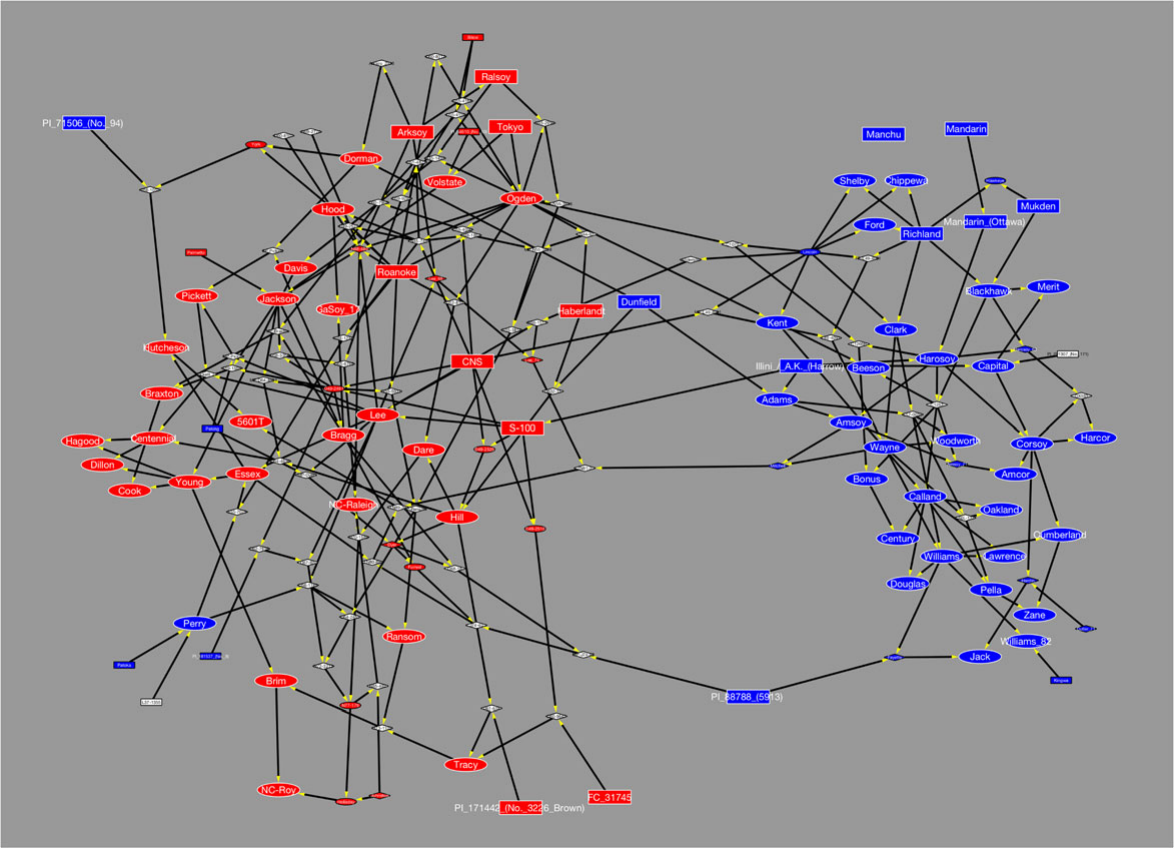
Separation of southern and northern genotypes. Pedigree data for all genotypes are shown as a directional network, in which soybean genotypes are represented as nodes and their relationship as edges. Edge points from parental lines to progeny lines as indicated by the *yellow arrowheads*. Landraces, milestone cultivars, and intermediate breeding lines are shown as *rectangles*, *ellipses*, and *diamonds*, respectively. *Nodes* shown in *blue* represent soybean lines belonging to maturity groups 0–IV, while *nodes* in *red* indicate lines with maturity ratings V–VIII. Genotypes whose maturity data were not available are shown as *white nodes*. Genotypes associated with large nodes surrounded by *white borders* were sequenced. The network analysis reveals two main clusters containing soybean lines adapted to more northern or southern growing zones (color figure online)

Cultivars with a wide range of maturity groups have been bred in North America since the first soybean hybrid cultivar was released. To associate soybean maturity with North American soybean pedigrees, we compiled pedigree and maturity group data of 166 soybean genotypes through comprehensive database and literature searches. These genotypes include landrace and milestone cultivars that represent 90 years of North American soybean breeding. The cultivars belong to diverse maturity groups (MG) from 0 to VIII. The pedigree data were analyzed and visualized using a networking approach (Shannon et al. 2003) (Fig. 1). A total of 166 soybean cultivars were represented as nodes and 274 parent-offspring relationships were represented as directed edges pointing from parental to progeny cultivars. The soybean cultivars grouped into two distinct clusters (Fig. 1). The smaller cluster contained 55 cultivars and 85 parent-offspring connections, and the larger cluster consisted of 110 cultivars with 180 parent-offspring relations. Only eight parent-offspring relations bridged the two clusters. Interestingly, the two clusters were defined by cultivars of either northern (MG 0–IV) or southern (MG V to VIII) maturity groups. Cultivars in the smaller cluster exclusively belonged to maturity groups 0–IV, while cultivars in the larger cluster predominantly belonged to maturity groups V–VIII. Only five of the 110 cultivars in the large southern cluster were northern cultivars. For example, Perry, a milestone cultivar in maturity group IV, was situated in the southern cluster. A small number of landrace and milestone cultivars had offspring in both clusters and thereby bridged them. Those cultivars were situated closer to the border between both clusters. For instance, Illini/A.K. (Harrow) (MG III) gave rise to Adams (MG III) in the northern cluster and S-100 (MG V) in the southern cluster, and Dunfield produced Adams in the northern cluster and Dorman in the southern cluster. The pedigree network analysis clearly demonstrated the separation of northern and southern breeding programs. This separation presumably limited genetic exchange between northern and southern cultivars and may have created distinct gene pools for southern and northern breeding programs respectively. Beneficial alleles, which are uniquely selected in southern or northern breeding program, could be integrated together by crossing southern and northern genotypes.

**Fig. 2:**
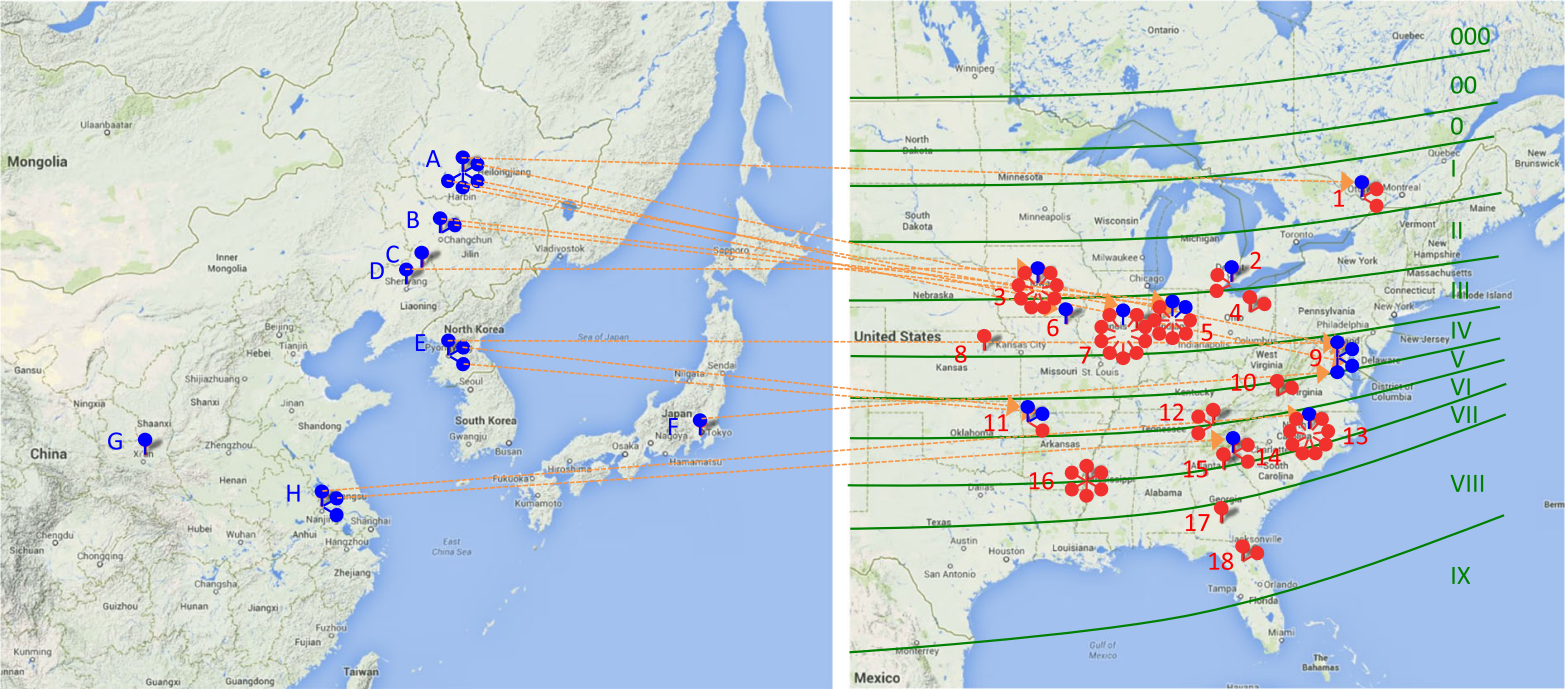
Geographic locations of origin and development of landraces and milestone cultivars. The geographic maps of East Asia and North America are in scale and aligned by latitude. Soybean maturity zones ranging from *000* to *IX* are superimposed on the map. *Letters* refer to locations of landrace collection in East Asia, and *numbers* indicate sites of landrace and/or milestone cultivar development in North America. Both are sorted by latitude from north to south. For few selected soybean varieties, *dashed lines* are shown connecting locations of origin with sites of introduction. *Blue dots* refer to landraces and *red dots* to milestone cultivars. Landraces (listed with maturity groups) were collected at following East Asian locations (country, province/city): *A* China, Heilongjiang: Illini (*III*), Manchu (*III*), Mandarin (Ottawa) (*0*), Mandarin (1), S-100 (*V*); *B* China, Jilin: Dunfield (*III*), Richland (*II*); *C* China, Liaoning: PI 88788 (*III*); *D* China, Liaoning: Mukden (*II*); *E* North Korea, Pyongyang: Arksoy (*VI*), Haberlandt (*VI*), Ralsoy (*VI*); *F* Japan, Kanagawa: Tokyo (*VII*); *G* China, Shaanxi: PI 171442 (*V*); *H* China, Jiangsu: CNS (*VII*), PI 71506 (*IV*), Roanoke (*VII*). Landraces and milestone cultivars (listed with maturity groups) were developed at following sites in North America (country, state/province, city): *1* Canada, Ontario, Ottawa: Merit (*0*), Capital (*0*), Mandarin (Ottawa) (*0*); *2* Canada, Ontario, Harrow: Harcor (*II*), Harosoy (*II*), A.K. (Harrow) (*III*); *3* USA, Iowa, Ames: Adams (*III*), Amsoy (*II*), Blackhawk (I), Corsoy (*II*), Cumberland (*III*), Ford (*III*), Oakland (*III*), Pella (*III*), Mukden (*II*); *4* USA, Ohio, Wooster: Amcor (*II*), Zane (*III*); *5* USA, Indiana, West Lafayette: Beeson (*II*), Bonus (*IV*), Calland (*III*), Century (*II*), Kent (*IV*), Perry (*IV*), Dunfield (*III*), Richland (*II*); *6* USA, Missouri, Rutledge: S-100 (*V*); *7* USA, Illinois, Urbana: Chippewa (I), Clark (*IV*), Jack (*II*), Lawrence (*IV*), Shelby (*III*), Wayne (*III*), Williams (*III*), Williams 82 (*III*), Woodworth (*III*), Illini (*III*); *8* USA, Kansas, Manhattan: Douglas (*IV*); *9* USA, Virginia, Arlington: Haberlandt (*VI*), Manchu (*III*); *10* USA, Virginia, Blacksburg: Essex (*V*), Hutcheson (*V*), Mandarin (I), Tokyo (*VII*); *11* USA, Arkansas, Fayetteville: Davis (*VI*), Arksoy (*VI*), Ralsoy (*VI*); *12* USA, Tennessee, Knoxville: 5601T (*V*), Ogden (*VI*), Volstate (*VII*); *13* USA, North Carolina, Raleigh: NC-Roy (*VI*), Brim (*VI*), Dare (*V*), Jackson (*VII*), NC-Raleigh (*VII*), Pickett (*VI*), Ransom (*VII*), Young (*VI*), Roanoke (*VII*); *14* USA, South Carolina, Clemson: Dillon (*VI*), Hagood (*VII*), CNS (*VII*); *15* USA, Georgia, Athens: Cook (*VIII*); *16* USA, Mississippi, Stoneville: Centennial (*VI*), Dorman (*V*), Hill (*V*), Hood (*VI*), Lee (*VI*), Tracy (*VI*); *17* USA, Georgia, Tifton: GaSoy17 (*VII*); *18* USA, Florida, Gainesville: Bragg (*VII*), Braxton (*VII*) (color figure online)

To understand genetic and molecular basis underlying maturity and flowering time variations of major cultivars, we selected 75 of the 166 genotypes for further analysis. The 75 genotypes represent historically and economically important landrace and milestone cultivars (Table 1). Forty cultivars have maturity groups (MG) 0 to IV, while 35 cultivars are assigned to maturity groups V to VIII. The landraces were collected in East Asia from a wide range of latitudes. They comprise 14 landraces from China, three from North Korea, one from Japan, and one from an unknown origin. Overall, landraces were preferentially introduced from Asia to locations of similar latitude in North America and were subsequently used to develop a variety of cultivars at these sites (Fig. 2). For about 70% of the landraces, the latitudes of collection and introduction sites differed by less than 3.7°. For example, Mandarin (Ottawa) originated in Heilongjiang, China and was introduced in Ontario, Canada. Likewise, Mukden was brought from Liaoning, China to Iowa, USA.

**Table 1:** List of landraces and milestone cultivars and their allelic variants at maturity genes *E1* to *E4*

Name^a^	Accession	Cultivar	Maturity group	*E1*	*E2*	*E3*	*E4*
Capital	PI 548311	Milestone	0	*e1-as*	*E2*	*e3*	*E4*
Mandarin (Ottawa)	PI 548379	Landrace	0	*e1-as*	e2	*e3*	*E4*
Merit	PI 548545	Milestone	0	*E1*	e2	*e3*	*E4*
Blackhawk	PI 548516	Milestone	I	*E1*	e2	*e3*	*E4*
Chippewa	PI 548530	Milestone	I	*e1-as*	e2	*e3*	*E4*
Mandarin	PI 548378	Landrace	I	*e1-as*	e2	*E3*	*E4*
Amcor	PI 548505	Milestone	II	*e1-as*	*E2*	*e3*	*E4*
Amsoy	PI 548506	Milestone	II	*e1-as*	e2	*E3*	*E4*
Beeson	PI 548510	Milestone	II	*E1*	e2	*E3*	*E4*
Century	PI 548512	Milestone	II	*e1-as*	e2	*E3*	*E4*
Corsoy	PI 548540	Milestone	II	*e1-as*	*E2*	*e3*	*E4*
Harcor	PI 548570	Milestone	II	*e1-as*	*E2*	*e3*	*E4*
Harosoy	PI 548573	Milestone	II	*e1-as*	e2	*E3*	*E4*
Jack	PI 540556	N/A	II	*e1-as*	*E2*	*E3*	*E4*
Mukden	PI 548391	Landrace	II	*E1*	e2	*E3*	*E4*
Richland	PI 548406	Landrace	II	*E1*	e2	*e3*	*E4*
5913	PI 88788	Landrace	III	*E1*	*E2*	*E3*	*E4*
A.K. (Harrow)	PI 548298	Landrace	III	*E1*	*E2*	*E3*	*E4*
Adams	PI 548502	Milestone	III	*E1*	e2	*E3*	*E4*
Calland	PI 548527	Milestone	III	*e1-as*	e2	*E3*	*E4*
Cumberland	PI 548542	Milestone	III	*e1-as*	*E2*	*E3*	*E4*
Dunfield	PI 548318	Landrace	III	*E1*	e2	*E3*	*E4*
Ford	PI 548562	Milestone	III	*e1-as*	*E2*	*E3*	*E4*
Illini	PI 548348	Landrace	III	*E1*	*E2*	*E3*	*E4*
Manchu	PI 548365	Landrace	III	*e1-as*	*E2*	*E3*	*E4*
Oakland	PI 548543	Milestone	III	*e1-as*	*E2*	*E3*	*E4*
Pella	PI 548523	Milestone	III	*e1-as*	e2	*E3*	*E4*
Shelby	PI 548574	Milestone	III	*e1-as*	*E2*	*E3*	*E4*
Wayne	PI 548628	Milestone	III	*e1-as*	*E2*	*E3*	*E4*
Williams	PI 548631	Milestone	III	*e1-as*	*E2*	*E3*	*E4*
Williams 82	PI 518671	Milestone	III	*e1-as*	*E2*	*E3*	*E4*
Woodworth	PI 548632	Milestone	III	*e1-as*	*E2*	*E3*	*E4*
Zane	PI 548634	Milestone	III	*e1-as*	*E2*	*E3*	*E4*
Bonus	PI 548517	Milestone	IV	*e1-as*	*E2*	*E3*	*E4*
Clark	PI 548533	Milestone	IV	*e1-as*	*E2*	*E3*	*E4*
Douglas	PI 548555	Milestone	IV	*e1-as*	*E2*	*E3*	*E4*
Kent	PI 548586	Milestone	IV	*e1-as*	*E2*	*E3*	*E4*
Lawrence	PI 518673	Milestone	IV	*e1-as*	*E2*	*E3*	*E4*
No. 94	PI 71506	Landrace	IV	*E1*	e2	*e3*	*E4*
Perry	PI 548603	Milestone	IV	*E1*	*E2*	*E3*	*E4*
5601 T	PI 630984	Milestone	V	*E1*	*E2*	*E3*	*E4*
Dare	PI 548987	Milestone	V	*E1*	*E2*	*E3*	*E4*
Dorman	PI 548653	Milestone	V	*E1*	*E2*	*e3*	*E4*
Essex	PI 548667	Milestone	V	*E1*	*E2*	*E3*	*E4*
Hill	PI 548654	Milestone	V	*E1*	*E2*	*E3*	*E4*
Hutcheson	PI 518664	Milestone	V	*E1*	*E2*	*E3*	*E4*
No. 3226 Brown	PI 171442	Landrace	V	*E1*	e2	*E3*	*E4*
S-100	PI 548488	Landrace	V	*E1*	*E2*	*E3*	*E4*
Arksoy	PI 548438	Landrace	VI	*E1*	*E2*	*e3*	*E4*
Brim	PI 548986	Milestone	VI	*E1*	*E2*	*E3*	*E4*
Centennial	PI 548975	Milestone	VI	*E1*	*E2*	*E3*	*E4*
Davis	PI 553039	Milestone	VI	*E1*	*E2*	*E3*	*E4*
Dillon	PI 592756	Milestone	VI	*E1*	*E2*	*E3*	*E4*
FC 31745	FC 31745	Landrace	VI	*E1*	*E2*	*E3*	*E4*
Haberlandt	PI 548456	Landrace	VI	*E1*	*E2*	*e3*	*E4*
Hood	PI 548980	Milestone	VI	*E1*	*E2*	*E3*	*E4*
Lee	PI 548656	Milestone	VI	*E1*	*E2*	*E3*	*E4*
NC-Roy	PI 617045	Milestone	VI	*E1*	*E2*	*E3*	*E4*
Ogden	PI 548477	Milestone	VI	*E1*	*E2*	*E3*	*E4*
Pickett	PI 548988	Milestone	VI	*E1*	*E2*	*E3*	*E4*
Ralsoy	PI 548484	Landrace	VI	*E1*	*E2*	*e3*	*E4*
Tracy	PI 548983	Milestone	VI	*E1*	*E2*	*E3*	*E4*
Young	PI 508266	Milestone	VI	*E1*	*E2*	*E3*	*E4*
Bragg	PI 548660	Milestone	VII	*E1*	*E2*	*E3*	*E4*
Braxton	PI 548659	Milestone	VII	*E1*	*E2*	*E3*	*E4*
CNS	PI 548445	Landrace	VII	*E1*	*E2*	*E3*	*E4*
GaSoy17	PI 553046	Milestone	VII	*E1*	*E2*	*E3*	*E4*
Hagood	PI 555453	Milestone	VII	*E1*	*E2*	*E3*	*E4*
Jackson	PI 548657	Milestone	VII	*E1*	*E2*	*E3*	*E4*
NC-Raleigh	PI 641156	Milestone	VII	*E1*	*E2*	*E3*	*E4*
Ransom	PI 548989	Milestone	VII	*E1*	*E2*	*E3*	*E4*
Roanoke	PI 548485	Landrace	VII	*E1*	*E2*	*E3*	*E4*
Tokyo	PI 548493	Landrace	VII	*E1*	*E2*	*E3*	*E4*
Volstate	PI 548494	Milestone	VII	*E1*	*E2*	*E3*	*E4*
Cook	PI 553045	Milestone	VIII	*E1*	*E2*	*E3*	*E4*

^a^Cultivars are sorted by maturity group

*N/A* not available

The divergence in flowering time and maturity between southern and northern genotypes likely represents one of the major factors leading to the two genetically separated breeding populations. Although more than 100 genes may be involved in flowering pathways in soybean (Kim et al. 2012), only ten loci (*E1–E9*, *J*) have been mapped and reported to control time to flowering and maturity. Maturity genes *E1*, *E2*, *E3*, and *E4* have been identified and sequenced (Liu et al. 2008; Tsubokura et al. 2013; Watanabe et al. 2012; Watanabe et al. 2009; Watanabe et al. 2011; Xia et al. 2012), and various soybean cultivars have been screened for their allelic variants (Langewisch et al. 2014; Tsubokura et al. 2014; Zhai et al. 2014). It has been estimated that the four maturity genes contribute between 62 and 66% of variation of flowering time in a population containing 63 soybean accessions (Tsubokura et al. 2014). We recently sequenced soybean seed transcriptomes of the 75 landraces and milestone cultivars at a seed mid-maturation stage, which provided us the opportunity to investigate molecular and genetic changes of the four maturity genes simultaneously in those cultivars. We determined the transcript sequence and expression levels of the *E1* to *E4* genes and/or associated the allelic variants with the maturity ratings of their soybean cultivars.

### Maturity gene *E2*

E2 has high homology to the Arabidopsis GIGANTEA protein, which is involved in the circadian clock of the flowering time pathway (Watanabe et al. 2011). E2 presumably controls the expression of the *Flowering Locus T* (*FT*) orthologs, which encode florigens (i.e., leaf-derived, mobile, long-distance signals promoting floral transition) (Watanabe et al. 2011). A nonsynonymous SNP in an *e2* allele has recently been reported where a thymine (T) was substituted for an adenine (A) resulting in a nonsense mutation (Watanabe et al. 2011). This premature stop codon truncates the E2 protein from 1170 amino acids to 521 amino acids, which lead to early flowering. We observed that *E2* (Glyma.10G221500) was highly expressed in seeds (Suppl. Fig. 1A). *E2* contained four SNPs in those genotypes, i.e., one synonymous SNP (chr10: 45,305,867), two nonsynonymous SNPs (chr10: 45,305,285, chr10: 45,310,798), and one SNP in the 3′ UTR (chr10: 45,315,921) (Suppl. Fig. 1A). The nonsynonymous SNP at chr10: 45,310,798 resulted in the previously reported premature stop codon and the production of the truncated nonfunctional E2 protein (Watanabe et al. 2011). This SNP was detected in 17 of the 75 examined cultivars (Table 2 and Suppl. Fig. 1A). With the exception of PI 171442, all cultivars carrying this nonsense mutation belonged to the northern maturity groups 0 to IV. Thus, this SNP represented an important functional allele accounting for some of the maturity variations in the landrace and milestone cultivars. However, none of the other three SNPs showed any significant correlation with maturity groups. Interestingly, we observed a lower expression of the *e2* mutant allele compared to the functional *E2* alleles. The average *e2* transcript accumulation was reduced to a level of 9.71 FPKM (Fragments Per Kilobase of transcript per Million mapped reads) from an average *E2* level of 21.99 FPKM. The decreased *e2* transcript accumulation might be caused by the premature stop codon through the nonsense-mediated mRNA decay (NMD) pathway (Merai et al. 2013).

**Table 2:** Summary of cultivars containing either *e1*, *e2*, or *e3* mutant allele

Gene	No. of northern cultivars MG 0–IV	No. of southern cultivars MG V–VIII	Total
*e1*	28/40	0/35	28/75
*e2*	16/40	1/35	17/75
*e3*	10/40	4/35	14/75

We determined the haplotype block structure containing the *E2* gene using the Haploview software package (Barrett et al. 2005). We identified three major haplotypes and one minor haplotype where *E2* was embedded in (Suppl. Fig. 1A, B). Haplotype 1 contained the *e2* mutant allele with the premature stop codon, while none of haplotypes 2, 3, and 4 did. Interestingly, haplotypes 1 and 3 were identical with the exception of the nonsense mutation. The seventeen cultivars carrying haplotype 1 included seven landraces (Mandarin (I), Mandarin (Ottawa) (0), Mukden (II), Richland (II), Dunfield (III), PI 71506 (IV), and PI 171442 (V)) collected from various regions in China, and ten milestone cultivars derived from those landraces (Adams (III), Blackhawk (I), Chippewa (I), Harosoy (II), Merit (0), Amsoy (II), Calland (III), Beeson (II), Century (II), and Pella (III)). Thus, the nonsense SNP allele in haplotype 1 has been widely present in ancestral landraces. It likely arose as a single nucleotide mutation in a common progenitor genotype carrying haplotype 3 (Table 1 and Suppl. Fig. 1A).

### Maturity gene *E3*

The *E3* gene (Glyma.19G224200) encodes a phytochrome A photoreceptor that affects the photoperiodic control of *FT2a* and *FT5a* expression and therefore flowering. Recently, a 13.3-kb deletion in an *e3* allele has been detected, which starts in intron 4 and includes the entire 3′ end of the gene (Watanabe et al. 2009). The deletion of the histidine kinase domain renders the E3 protein nonfunctional, which results in an early flowering phenotype. A nonfunctional *e3* allele containing a 2.6-kb transposon insertion in intron 4 and a nonsynonymous SNP (G1050R) in exon 3 has been described as well (Shin and Lee 2012; Watanabe et al. 2009). We observed that the *E3* gene is only weakly expressed in soybean seeds at a mean level of 0.93 FPKM with little variation in the examined cultivars. In addition, *E3* had no SNPs in the regions sequenced in all cultivars. However, inspection of the short sequencing read alignments to the genomic reference sequence using the Integrative Genomics Viewer (IGV) revealed a large deletion in 14 of 75 soybean cultivars (Suppl. Fig. 2A) (Robinson et al. 2011; Thorvaldsdóttir et al. 2013). The deletion is likely identical with the 13.3-kb deletion previously reported in the *E3* gene that results in an early flowering phenotype (Watanabe et al. 2009). The deletion also starts in intron 4 and probably includes the adjacent gene model Glyma.19G224300, which is not expressed in *e3* mutants and about 7.3 kb apart from exon 4 of *e3* (Suppl. Fig. 2A). Interestingly, a number of sequencing reads contained the splice junction of exon 4 from *e3* (Glyma.19G224200) and exon 2 from Glyma.19G224400, which are 18 kb apart in the Williams 82 reference genome, suggesting that transcription cross the deletion junction into Glyma.19G224400, followed by splicing of the novel intron. Therefore, the deletion generated a chimeric transcript consisting of the truncated *e3* allele and Glyma.19G224400. The *e3* deletion was present in six landraces (Arksoy (VI), Ralsoy (VI), Haberlandt (VI), Mandarin (Ottawa) (0), PI 71506 (IV), and Richland (II)) and eight milestone lines (Capital (0), Blackhawk (I), Chippewa (I), Dorman (V), Merit (0), Amcor (II), Corsoy (II), and Harcor (II)) (Table 1 and Suppl. Fig. 2A). They belong to maturity groups ranging from 0 to VI. The *e3* mutant landraces were collected in various regions in China and North Korea, which indicate the wide distribution of the *e3* mutant allele. In addition, we identified six haplotypes containing the *E3* gene, which spanned about 213 kb (Suppl. Fig. 2B, C). The *e3* deletion allele was located in haplotype 1. The predominant haplotype 6 was found in cultivars with maturity groups from I to VIII, while haplotype 3 was associated with southern maturity groups V to VII. The remaining haplotypes 2, 4, and 5 are rare, as none of them were present in more than three cultivars (Suppl. Fig. 2B).

### Maturity gene *E4*

Similar to *E3*, *E4* (Glyma.20G090000) also encodes a phytochrome A (phyA) photoreceptor, which controls the *Flowering Locus T* orthologs *FT2a* and *FT5a* (Liu et al. 2008; Tsubokura et al. 2013). Five nonfunctional alleles have been reported. They are caused by one 6.2-kb retroelement insertion in exon 1 (*e4* (*SORE-1*)) and four 1-bp deletions (*e4-oto*, *e4-tsu*, *e4-kam*, *e4-kes*) in the coding region creating frameshifts, premature stop codons, and truncated proteins (Liu et al. 2008; Tsubokura et al. 2013). *E4* was expressed in mid-maturation seeds at mean FPKM levels of 3.21. Although various *e4* mutant alleles have been identified previously, we did not detect any SNPs, small indels, or significant expression variation among our 75 cultivars. Neither did we find larger deletions or insertion upon visual inspection of sequencing read alignments, suggesting that there is no obvious genetic variation of *E4* among the 75 genotypes. Consequently, *E4* does not seem to contribute to the maturity variation of those landrace and milestone cultivars. *E4* cannot be assigned to a haplotype block either.

### Maturity gene *E1*

*E1* encodes a putative transcription factor containing a plant-specific B3 domain. E1 inhibits the floral induction under long-day growth conditions as it suppresses the expression of the *Flowering Locus T* orthologs *FT2a* and *FT5a*. The expression of *E1* is under the photoperiodic control of E3 and E4 (Xu et al. 2015). Several nonfunctional *e1* alleles have been identified. *e1-fs* allele has a 1-bp deletion causing a frameshift, and *e1-nl* is a null allele with a deletion of the entire *E1* gene. A missense point mutation at nucleotide 44 in the nuclear localization signal of the *e1-as* allele results in a dysfunctional protein and early flowering (Xia et al. 2012). In contrast to *E2*, *E3*, and *E4*, we did not detect any expression of *E1* (Glyma.06G207800) in seeds. However, we identified a haplotype block that contained the *E1* gene in five distinct haplotypes among the examined cultivars (Suppl. Fig. 3). Williams 82 carries the recessive *e1-as* mutant allele (Xia et al. 2012). Twenty-seven cultivars revealed the same haplotype 1 as Williams 82 (Table 1 and Suppl. Fig. 3), suggesting that they may carry the same *e1-as* allele. Three landraces (Mandarin, Mandarin (Ottawa), and Manchu) were among the 27 cultivars. Interestingly, all landraces that gave rise to the putative 25 *e1-as* milestone cultivars were collected in Heilongjiang, a region in Northeast China (Fig. 2), indicating that the *e1-as* allele may have originated in Heilongjiang. The 28 presumably *e1-as* cultivars belonged to maturity groups 0 to IV, which accounted for 70% of the examined 40 northern cultivars (Suppl. Fig. 3 and Table 2).

The *e1-as* allele represented the most predominant *e* mutant allele among our examined North American cultivars, followed by *e2* and then *e3* (Table 2). *E4* unlikely contributed to the maturity variations of the landrace and milestone cultivars. The *e1-as* haplotype was only detected in northern cultivars and not in any southern cultivar. However, one *e2* allele and four *e3* alleles have been identified within southern genotypes (Table 2). Our results support the previous hypthesis that *E1* has the strongest and *E3* the weakest effect on flowering time among the *E1*, *E2*, and *E3* genes (Tsubokura et al. 2014). However, those mutant alleles likely have additive and combinatorial effects. Double mutant cultivars with *e1/e2* (MG I to III), *e1/e3* (MG 0 to II), and *e2/e3* (MG 0 to IV) alleles exclusively belong to northern maturity groups (Table 3). Triple mutant cultivars, i.e., Mandarin (Ottawa) and Chippewa, are in maturity groups 0 and I, respectively. Interestingly, cultivars containing the same allelic combinations could differ dramatically in their maturity rating. The allelic variations and their combinations did not entirely correlate with maturity ratings of the landrace and milestone cultivars. In addition, none of four northern genotypes PI 88788, Illini, A.K. (Harrow), and Perry contained any of the *e1*, *e2*, or *e3* mutant alleles (Table 1 and Table 3). Thus, it is likely that allelic variations at additional maturity loci are present in those landrace and milestone cultivars. Our observation is consistent with an earlier study of different soybean cultivars, in which only 62 to 66% of variation of flowering time could be explained by the *E1* to *E4* maturity genes (Tsubokura et al. 2014).

**Table 3:** Summary of cultivars containing *e1*, *e2*, or *e3* single, double, or triple mutant alleles

*E1*	*E2*	*E3*	No. of northern cultivars MG 0–IV	No. of southern cultivars MG V–VIII	MG range
*e1-as*	e2	*e3*	2	0	0–I
*e1-as*	e2	*E3*	6	0	I–III
*e1-as*	*E2*	*E3*	16	0	II–IV
*e1-as*	*E2*	*e3*	4	0	0–II
*E1*	e2	*e3*	4	0	0–IV
*E1*	e2	*E3*	4	1	II–V
*E1*	*E2*	*e3*	0	4	V–VI
*E1*	*E2*	*E3*	4	30	III–VIII

## Electronic supplementary material

Supplemental Figure 1A E2 alleles and haplotypes. Haplotypes containing the E2 maturity gene are displayed for each of the 75 landraces and milestone cultivars. Three major and one minor haplotype can be distinguished. The first major haplotype carries the e2 mutant allele and coincides with mostly northern maturity ratings. The second major haplotype is present in both northern and southern lines while the third major haplotype is associated with southern maturity groups. The SNP (A in E2 and T in e2) causing the non-sense mutation in e2 is framed in red. The length of the haplotype block has been manually adjusted. All cultivars are sorted by haplotype and maturity group, both of which are noted next to the line designation. Cultivars in maturity groups 0 to IV are written in black, while cultivars in maturity groups V to VIII are written in red. Major and minor SNP alleles are shown in red and black, respectively. Individual expression values are listed for each cultivar. Only highly reliable SNP positions that are present in all cultivars are used in this analysis. Their chromosomal positions and gene models in which they are located are indicated. (PDF 50 kb).

Supplemental Figure 1B LD plot and Haplotype block containing the E2 gene. The E2 gene is located in haplotype block 76. The haplotype containing the e2 mutant allele is framed in red. The SNP position that is associated with the non-sense mutation is also framed in red. The corresponding LD plot is displayed below the haplotype blocks. (PDF 107 kb).

Supplemental Figure 2A IGV view of the e3 mutant allele. The IGV view of E3 and two downstream genes suggests a large deletion that contains the 3’ end of E3 and the adjacent gene model Glyma.19G224300. While Williams 82 shows the expected expression pattern of E3 as indicated in the gene model panel at the bottom of the figure, six landraces and eight milestone cultivars reveal no expression after exon 4. (PDF 919 kb).

Supplemental Figure 2B: E3 haplotypes. The entire haplotype block that includes the E3 gene is about 213 kb in size. Six haplotypes can be identified. The e3 mutant allele is located in haplotype 1. E3 does not contain SNPs but is shown between neighboring SNPs. The haplotype block is presented in the same format as in Suppl. Figure 1A (see Suppl. Figure legend 1A for more information). (PDF 56 kb).

Supplemental Figure 2C: LD plot and Haplotype block containing the E3 gene. The E3 gene is located in haplotype block 93. The haplotype containing the e3 mutant is framed in red. The E3 gene does not have SNPs, but the E3 position is shown with a triangle pointing in between two SNPs adjacent to E3. The corresponding LD plot is displayed below the haplotype blocks. (PDF 235 kb).

Supplemental Figure 3 E1 haplotypes. Six haplotypes containing the E1 gene are presented here. The e1 mutant allele is situated in haplotype 1. E1 is not expressed in seeds, therefore sequence or expression polymorphisms among our lines are not available for direct genotyping. The E1 gene is framed in red at the proper chromosomal position in the SNP table. Note that Williams 82, which shares haplotype 1 with 27 additional landraces and milestone varieties, carries the *e1-as* mutant allele. The haplotypes are presented in the same format as in Suppl. Figure 1A (see legend of Suppl. Figure 1A for more information). (PDF 50 kb).
