# Supplementary material for: Genetic separation of southern and northern soybean breeding programs in North America and their associated allelic variation at four maturity loci
Source: Mol Breed. 2017 Jan 11;37(1):8. doi: 10.1007/s11032-016-0611-7 (PMC5226990; doi:10.1007/s11032-016-0611-7)
Supplement: Supplementary file 1 — E2 alleles and haplotypes. Haplotypes containing the E2 maturity gene are displayed for each of the 75 landraces and milestone cultivars. Three major and one minor haplotype can be distinguished. The first major haplotype carries the e2 mutant allele and coincides with mostly northern maturity ratings. The second major haplotype is present in both northern and southern lines while the third major haplotype is associated with southern maturity groups. The SNP (A in E2 and T in e2) causing the non-sense mutation in e2 is framed in red. The length of the haplotype block has been manually adjusted. All cultivars are sorted by haplotype and maturity group, both of which are noted next to the line designation. Cultivars in maturity groups 0 to IV are written in black, while cultivars in maturity groups V to VIII are written in red. Major and minor SNP alleles are shown in red and black, respectively. Individual expression values are listed for each cultivar. Only highly reliable SNP positions that are present in all cultivars are used in this analysis. Their chromosomal positions and gene models in which they are located are indicated. (PDF 50 kb) [file 11032_2016_611_MOESM1_ESM.pdf]

cultivar	maturity group	haplotype	Glyma.10G221400	*E2*: Glyma.10G221500				Glyma.10G221800				*E2* FPKM	average *E2* FPKM
			chr10: 45,283,870	chr10: 45,305,285	chr10: 45,305,867	chr10: 45,310,798	chr10: 45,315,921	chr10: 45,329,450	chr10: 45,331,070	chr10: 45,331,299	chr10: 45,331,446		
Mandarin (Ottawa)	0	1	C	G	T	T	T	A	C	C	T	8.65	9.71 +/ 2.43
Merit	0		C	G	T	T	T	A	C	C	T	13.98	
Blackhawk	I		C	G	T	T	T	A	C	C	T	8.48	
Chippewa	I		C	G	T	T	T	A	C	C	T	13.44	
Mandarin	I		C	G	T	T	T	A	C	C	T	6.50	
Amsoy	II		C	G	T	T	T	A	C	C	T	7.57	
Beeson	II		C	G	T	T	T	A	C	C	T	12.92	
Century	II		C	G	T	T	T	A	C	C	T	9.22	
Harosoy	II		C	G	T	T	T	A	C	C	T	9.37	
Mukden	II		C	G	T	T	T	A	C	C	T	6.11	
Richland	II		C	G	T	T	T	A	C	C	T	8.71	
Adams	III		C	G	T	T	T	A	C	C	T	9.90	
Calland	III		C	G	T	T	T	A	C	C	T	9.09	
Dunfield	III		C	G	T	T	T	A	C	C	T	10.21	
Pella	III		C	G	T	T	T	A	C	C	T	7.52	
PI 71506	IV		C	G	T	T	T	A	C	C	T	9.84	
PI 171442	V		C	G	T	T	T	A	C	C	T	13.55	
Capital	0	2	T	A	G	A	C	G	G	T	C	24.81	21.99 +/- 3.77
Amcor	II		T	A	G	A	C	G	G	T	C	24.58	
Corsoy	II		T	A	G	A	C	G	G	T	C	14.96	
Harcor	II		T	A	G	A	C	G	G	T	C	17.67	
Jack	II		T	A	G	A	C	G	G	T	C	17.66	
PI 88788	III		T	A	G	A	C	G	G	T	C	29.71	
Cumberland	III		T	A	G	A	C	G	G	T	C	17.78	
Ford	III		T	A	G	A	C	G	G	T	C	22.72	
Illini / A.K. (Harrow)	III		T	A	G	A	C	G	G	T	C	20.00	
Manchu	III		T	A	G	A	C	G	G	T	C	22.82	
Oakland	III		T	A	G	A	C	G	G	T	C	18.37	
Shelby	III		T	A	G	A	C	G	G	T	C	21.88	
Wayne	III		T	A	G	A	C	G	G	T	C	24.74	
Williams	III		T	A	G	A	C	G	G	T	C	19.58	
Williams 82	III		T	A	G	A	C	G	G	T	C	21.12	
Woodworth	III		T	A	G	A	C	G	G	T	C	25.90	
Zane	III		T	A	G	A	C	G	G	T	C	27.46	
Clark	IV		T	A	G	A	C	G	G	T	C	22.92	
Douglas	IV		T	A	G	A	C	G	G	T	C	21.74	
Lawrence	IV		T	A	G	A	C	G	G	T	C	26.21	
Perry	IV		T	A	G	A	C	G	G	T	C	19.82	
5601T	V		T	A	G	A	C	G	G	T	C	15.72	
Essex	V		T	A	G	A	C	G	G	T	C	18.38	
Hill	V		T	A	G	A	C	G	G	T	C	19.71	
S-100	V		T	A	G	A	C	G	G	T	C	26.46	
Brim	VI		T	A	G	A	C	G	G	T	C	22.97	
Centennial	VI		T	A	G	A	C	G	G	T	C	23.38	
FC 31745	VI		T	A	G	A	C	G	G	T	C	9.32	
Haberlandt	VI		T	A	G	A	C	G	G	T	C	21.28	
Lee	VI		T	A	G	A	C	G	G	T	C	20.08	
NC-Roy	VI		T	A	G	A	C	G	G	T	C	21.50	
Pickett	VI		T	A	G	A	C	G	G	T	C	19.91	
Tracy	VI		T	A	G	A	C	G	G	T	C	23.36	
Bragg	VII		T	A	G	A	C	G	G	T	C	19.59	
Braxton	VII		T	A	G	A	C	G	G	T	C	20.89	
GaSoy17	VII		T	A	G	A	C	G	G	T	C	24.21	
Cook	VIII		T	A	G	A	C	G	G	T	C	19.70	
Bonus	IV	3	C	G	T	A	T	A	C	C	T	29.23	
Kent	IV		C	G	T	A	T	A	C	C	T	20.31	
Dare	V		C	G	T	A	T	A	C	C	T	20.15	
Dorman	V		C	G	T	A	T	A	C	C	T	27.74	
Hutcheson	V		C	G	T	A	T	A	C	C	T	21.65	
Arksoy	VI		C	G	T	A	T	A	C	C	T	19.51	
Davis	VI		C	G	T	A	T	A	C	C	T	28.91	
Dillon	VI		C	G	T	A	T	A	C	C	T	22.18	
Hood	VI		C	G	T	A	T	A	C	C	T	25.82	
Ogden	VI		C	G	T	A	T	A	C	C	T	26.39	
Ralsoy	VI		C	G	T	A	T	A	C	C	T	19.61	
Young	VI		C	G	T	A	T	A	C	C	T	25.35	
Hagood	VII		C	G	T	A	T	A	C	C	T	16.94	
Jackson	VII		C	G	T	A	T	A	C	C	T	20.28	
NC-Raleigh	VII		C	G	T	A	T	A	C	C	T	23.96	
Ransom	VII		C	G	T	A	T	A	C	C	T	24.89	
Roanoke	VII		C	G	T	A	T	A	C	C	T	21.71	
Tokyo	VII		C	G	T	A	T	A	C	C	T	22.88	
Volstate	VII		C	G	T	A	T	A	C	C	T	24.97	
CNS	VII	4	T	G	T	A	T	A	C	C	T	21.79	
