# Supplementary figures and images for: Genetic separation of southern and northern soybean breeding programs in North America and their associated allelic variation at four maturity loci
Source: Mol Breed. 2017 Jan 11;37(1):8. doi: 10.1007/s11032-016-0611-7 (PMC5226990; doi:10.1007/s11032-016-0611-7)

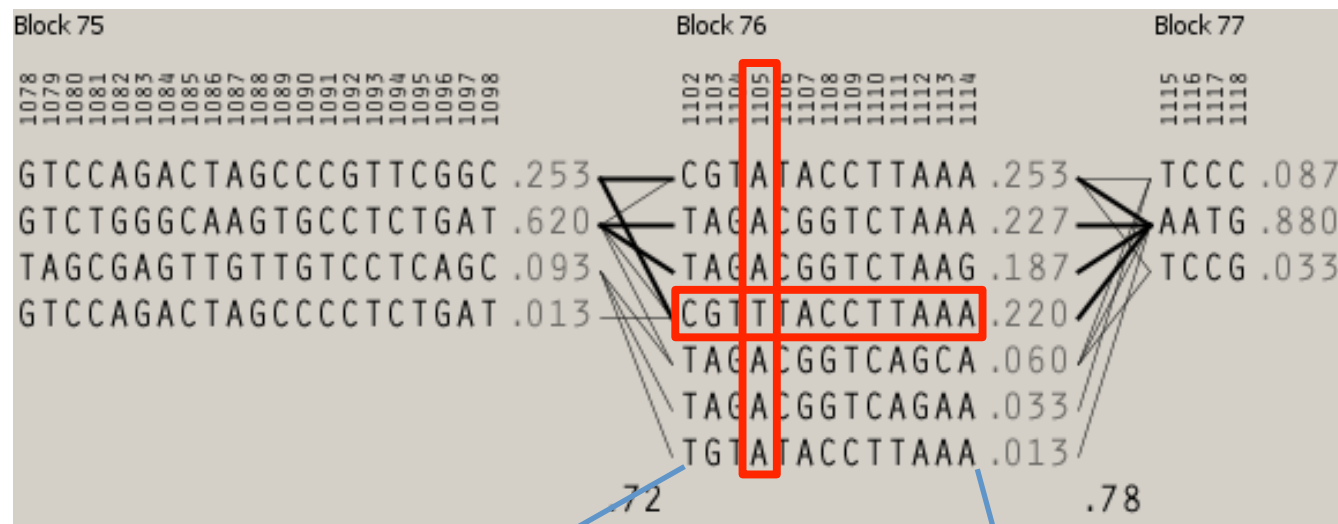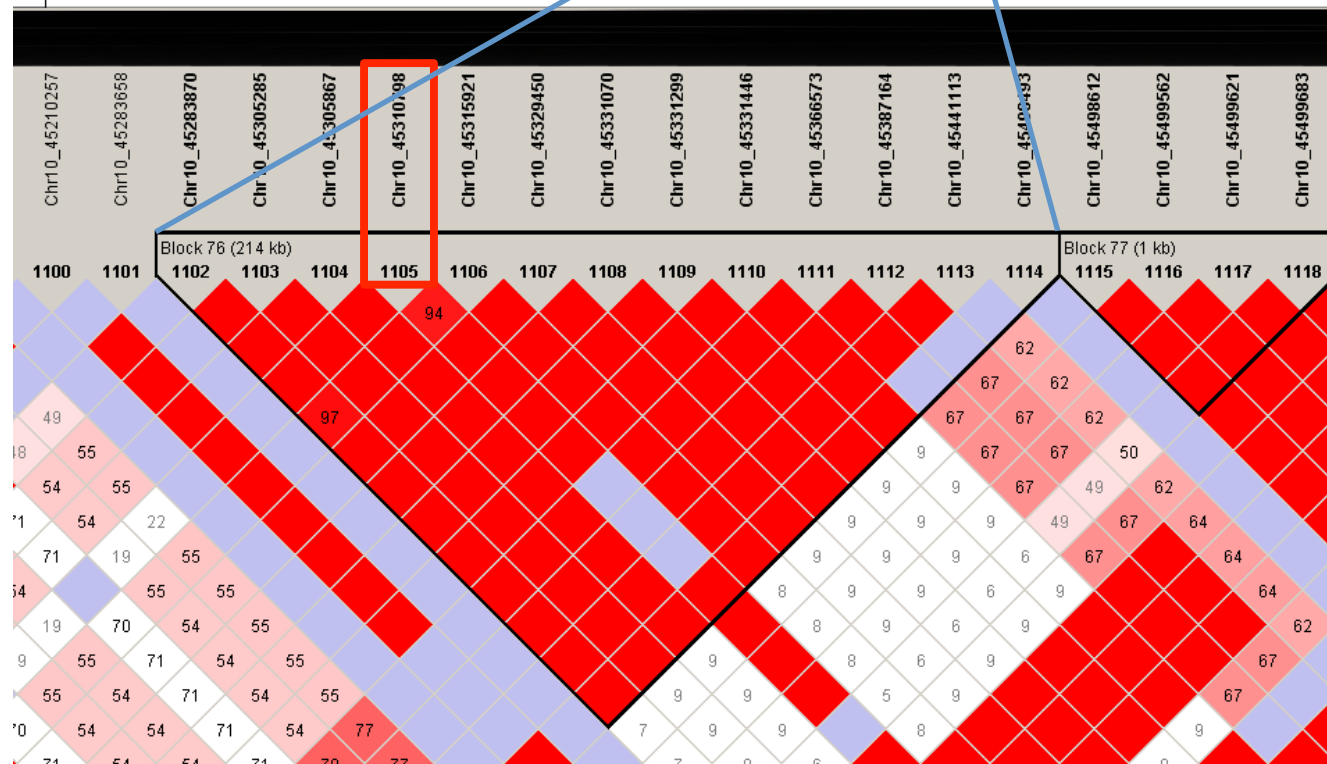

Supplement: Supplementary file 2 — LD plot and Haplotype block containing the E2 gene. The E2 gene is located in haplotype block 76. The haplotype containing the e2 mutant allele is framed in red. The SNP position that is associated with the non-sense mutation is also framed in red. The corresponding LD plot is displayed below the haplotype blocks. (PDF 107 kb) [file 11032_2016_611_MOESM2_ESM.pdf]

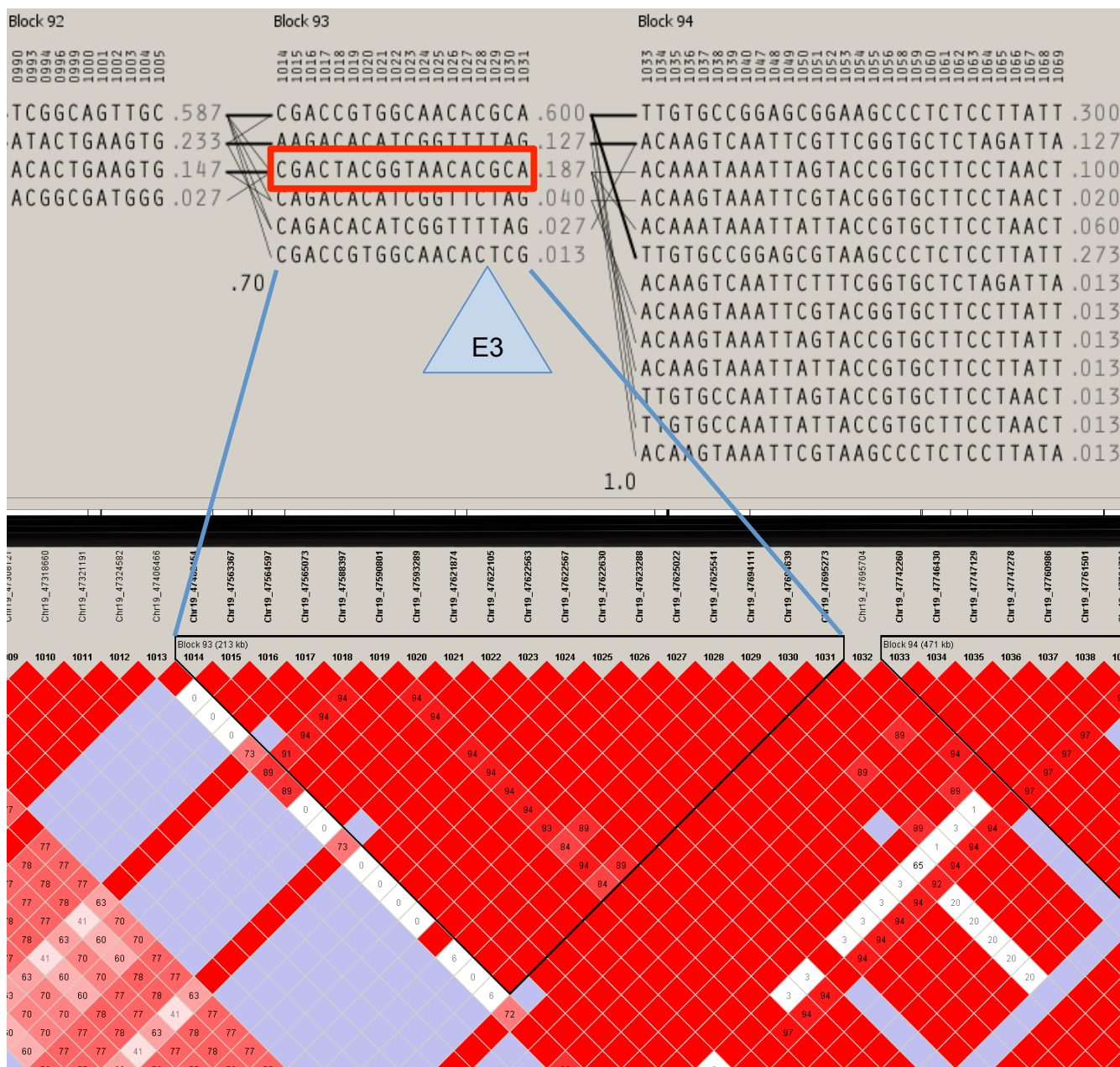

Supplement: Supplementary file 5 — LD plot and Haplotype block containing the E3 gene. The E3 gene is located in haplotype block 93. The haplotype containing the e3 mutant is framed in red. The E3 gene does not have SNPs, but the E3 position is shown with a triangle pointing in between two SNPs adjacent to E3. The corresponding LD plot is displayed below the haplotype blocks. (PDF 235 kb) [file 11032_2016_611_MOESM5_ESM.pdf]
