# Supplementary material for: Genetic separation of southern and northern soybean breeding programs in North America and their associated allelic variation at four maturity loci
Source: Mol Breed. 2017 Jan 11;37(1):8. doi: 10.1007/s11032-016-0611-7 (PMC5226990; doi:10.1007/s11032-016-0611-7)
Supplement: Supplementary file 3 — IGV view of the e3 mutant allele. The IGV view of E3 and two downstream genes suggests a large deletion that contains the 3’ end of E3 and the adjacent gene model Glyma.19G224300. While Williams 82 shows the expected expression pattern of E3 as indicated in the gene model panel at the bottom of the figure, six landraces and eight milestone cultivars reveal no expression after exon 4. (PDF 919 kb) [file 11032_2016_611_MOESM3_ESM.pdf]

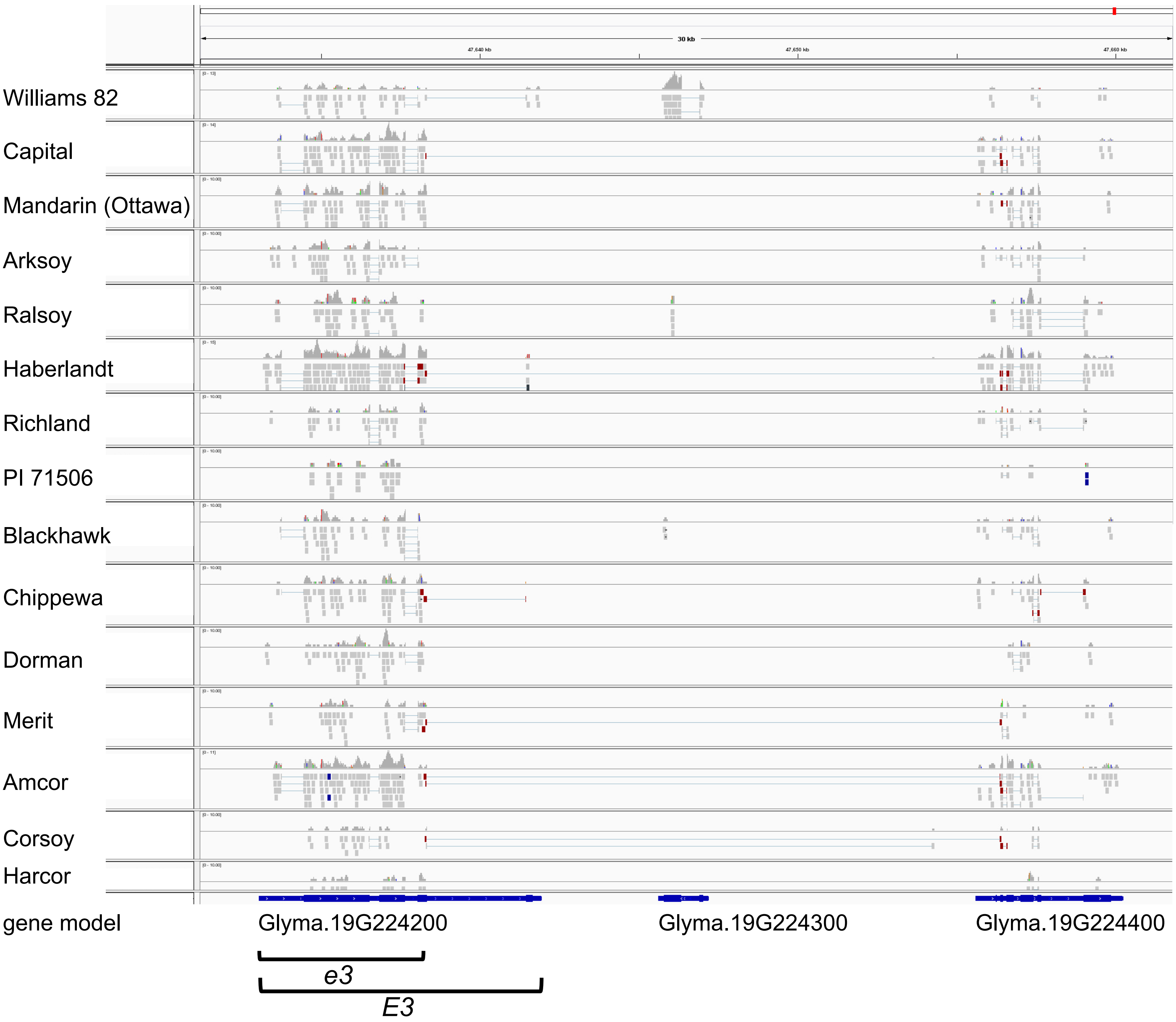

30 kb
47,640 kb
47,650 kb
47,660 kb
Williams 82
Capital
Mandarin (Ottawa)
Arksoy
Ralsoy
Haberlandt
Richland
PI 71506
Blackhawk
Chippewa
Dorman
Merit
Amcor
Corsoy
Harcor
gene model
Glyma.19G224200
Glyma.19G224300
Glyma.19G224400
e3
E3
