# Supplementary material for: Genetic separation of southern and northern soybean breeding programs in North America and their associated allelic variation at four maturity loci
Source: Mol Breed. 2017 Jan 11;37(1):8. doi: 10.1007/s11032-016-0611-7 (PMC5226990; doi:10.1007/s11032-016-0611-7)
Supplement: Supplementary file 4 — E3 haplotypes. The entire haplotype block that includes the E3 gene is about 213 kb in size. Six haplotypes can be identified. The e3 mutant allele is located in haplotype 1. E3 does not contain SNPs but is shown between neighboring SNPs. The haplotype block is presented in the same format as in Suppl. Figure 1A (see Suppl. Figure legend 1A for more information). (PDF 56 kb) [file 11032_2016_611_MOESM4_ESM.pdf]

			Glyma.19G222600	Glyma.19G223300			Glyma.19G223600			Glyma.19G224000								*E3*: Glyma.19G224200	Glyma.19G224700				
Name	maturity group	haplotype	chr19: 47,482,154	chr19: 47,563,367	chr19: 47,564,597	chr19: 47,565,073	chr19: 47,588,397	chr19: 47,590,801	chr19: 47,593,289	chr19: 47,621,874	chr19: 47,622,105	chr19: 47,622,563	chr19: 47,622,567	chr19: 47,622,630	chr19: 47,623,288	chr19: 47,625,022	chr19: 47,625,541	chr19: 47,633,059..47,641,958	chr19: 47,694,111	chr19: 47,694,639	chr19: 47,695,273	*E3* FPKM	average *E3* FPKM
Capital	0		C	G	A	C	T	A	C	G	G	T	A	A	C	A	C	*e3*	G	C	A	0.54	
Mandarin (Ottawa)	0		C	G	A	C	T	A	C	G	G	T	A	A	C	A	C	*e3*	G	C	A	0.99	
Merit	0		C	G	A	C	T	A	C	G	G	T	A	A	C	A	C	*e3*	G	C	A	0.46	
Blackhawk	I		C	G	A	C	T	A	C	G	G	T	A	A	C	A	C	*e3*	G	C	A	0.72	
Chippewa	I		C	G	A	C	T	A	C	G	G	T	A	A	C	A	C	*e3*	G	C	A	0.76	
Richland	II		C	G	A	C	T	A	C	G	G	T	A	A	C	A	C	*e3*	G	C	A	0.54	
Amcor	II	1	C	G	A	C	T	A	C	G	G	T	A	A	C	A	C	*e3*	G	C	A	0.88	0.71 +/ 0.24
Corsoy	II		C	G	A	C	T	A	C	G	G	T	A	A	C	A	C	*e3*	G	C	A	0.35	
Harcor	II		C	G	A	C	T	A	C	G	G	T	A	A	C	A	C	*e3*	G	C	A	0.50	
PI 71506	IV		C	G	A	C	T	A	C	G	G	T	A	A	C	A	C	*e3*	G	C	A	0.79	
Dorman	V		C	G	A	C	T	A	C	G	G	T	A	A	C	A	C	*e3*	G	C	A	1.07	
Haberlandt	VI		C	G	A	C	T	A	C	G	G	T	A	A	C	A	C	*e3*	G	C	A	1.09	
Arksoy	VI		C	G	A	C	T	A	C	G	G	T	A	A	C	A	C	*e3*	G	C	A	0.43	
Ralsoy	VI		C	G	A	C	T	A	C	G	G	T	A	A	C	A	C	*e3*	G	C	A	0.82	
Perry	IV		C	A	G	A	C	A	C	A	T	C	G	G	T	T	C	*E3*	T	A	G	1.23	
FC 31745	VI	2	C	A	G	A	C	A	C	A	T	C	G	G	T	T	C	*E3*	T	A	G	0.88	
Volstate	VII		C	A	G	A	C	A	C	A	T	C	G	G	T	T	C	*E3*	T	A	G	0.69	
Essex	V		A	A	G	A	C	A	C	A	T	C	G	G	T	T	T	*E3*	T	A	G	1.32	
Hill	V		A	A	G	A	C	A	C	A	T	C	G	G	T	T	T	*E3*	T	A	G	1.48	
PI 171442	V		A	A	G	A	C	A	C	A	T	C	G	G	T	T	T	*E3*	T	A	G	1.46	
Tracy	VI		A	A	G	A	C	A	C	A	T	C	G	G	T	T	T	*E3*	T	A	G	0.65	
Dillon	VI		A	A	G	A	C	A	C	A	T	C	G	G	T	T	T	*E3*	T	A	G	1.36	
Lee	VI	3	A	A	G	A	C	A	C	A	T	C	G	G	T	T	T	*E3*	T	A	G	1.60	
Pickett	VI		A	A	G	A	C	A	C	A	T	C	G	G	T	T	T	*E3*	T	A	G	0.78	
Young	VI		A	A	G	A	C	A	C	A	T	C	G	G	T	T	T	*E3*	T	A	G	0.68	
CNS	VII		A	A	G	A	C	A	C	A	T	C	G	G	T	T	T	*E3*	T	A	G	0.71	
Tokyo	VII		A	A	G	A	C	A	C	A	T	C	G	G	T	T	T	*E3*	T	A	G	0.90	
Hagood	VII		A	A	G	A	C	A	C	A	T	C	G	G	T	T	T	*E3*	T	A	G	1.25	
Hood	VI	4	C	A	G	A	C	A	C	A	T	C	G	G	T	T	T	*E3*	T	A	G	1.49	
Mukden	II	5	C	G	A	C	C	G	T	G	G	C	A	A	C	A	C	*E3*	T	C	G	0.62	
Mandarin	I		C	G	A	C	C	G	T	G	G	C	A	A	C	A	C	*E3*	G	C	A	0.83	
Amsoy	II		C	G	A	C	C	G	T	G	G	C	A	A	C	A	C	*E3*	G	C	A	0.76	
Beeson	II		C	G	A	C	C	G	T	G	G	C	A	A	C	A	C	*E3*	G	C	A	0.43	
Century	II		C	G	A	C	C	G	T	G	G	C	A	A	C	A	C	*E3*	G	C	A	0.68	
Harosoy	II		C	G	A	C	C	G	T	G	G	C	A	A	C	A	C	*E3*	G	C	A	0.61	
Jack	II		C	G	A	C	C	G	T	G	G	C	A	A	C	A	C	*E3*	G	C	A	0.89	
Dunfield	III		C	G	A	C	C	G	T	G	G	C	A	A	C	A	C	*E3*	G	C	A	0.76	
Illini / A.K. (Harrow)	III		C	G	A	C	C	G	T	G	G	C	A	A	C	A	C	*E3*	G	C	A	0.33	
Manchu	III		C	G	A	C	C	G	T	G	G	C	A	A	C	A	C	*E3*	G	C	A	1.38	
PI 88788	III		C	G	A	C	C	G	T	G	G	C	A	A	C	A	C	*E3*	G	C	A	0.65	
Adams	III		C	G	A	C	C	G	T	G	G	C	A	A	C	A	C	*E3*	G	C	A	0.56	
Calland	III		C	G	A	C	C	G	T	G	G	C	A	A	C	A	C	*E3*	G	C	A	1.12	
Cumberland	III		C	G	A	C	C	G	T	G	G	C	A	A	C	A	C	*E3*	G	C	A	1.12	
Ford	III		C	G	A	C	C	G	T	G	G	C	A	A	C	A	C	*E3*	G	C	A	1.01	0.98 +/ 0.33
Oakland	III		C	G	A	C	C	G	T	G	G	C	A	A	C	A	C	*E3*	G	C	A	1.19	
Pella	III		C	G	A	C	C	G	T	G	G	C	A	A	C	A	C	*E3*	G	C	A	0.98	
Shelby	III		C	G	A	C	C	G	T	G	G	C	A	A	C	A	C	*E3*	G	C	A	1.25	
Wayne	III		C	G	A	C	C	G	T	G	G	C	A	A	C	A	C	*E3*	G	C	A	1.51	
Williams	III		C	G	A	C	C	G	T	G	G	C	A	A	C	A	C	*E3*	G	C	A	0.77	
Williams 82	III		C	G	A	C	C	G	T	G	G	C	A	A	C	A	C	*E3*	G	C	A	0.77	
Woodworth	III		C	G	A	C	C	G	T	G	G	C	A	A	C	A	C	*E3*	G	C	A	1.39	
Zane	III	6	C	G	A	C	C	G	T	G	G	C	A	A	C	A	C	*E3*	G	C	A	0.73	
Bonus	IV		C	G	A	C	C	G	T	G	G	C	A	A	C	A	C	*E3*	G	C	A	1.01	
Clark	IV		C	G	A	C	C	G	T	G	G	C	A	A	C	A	C	*E3*	G	C	A	1.45	
Douglas	IV		C	G	A	C	C	G	T	G	G	C	A	A	C	A	C	*E3*	G	C	A	1.27	
Kent	IV		C	G	A	C	C	G	T	G	G	C	A	A	C	A	C	*E3*	G	C	A	1.63	
Lawrence	IV		C	G	A	C	C	G	T	G	G	C	A	A	C	A	C	*E3*	G	C	A	1.29	
Hutcheson	V		C	G	A	C	C	G	T	G	G	C	A	A	C	A	C	*E3*	G	C	A	0.81	
5601T	V		C	G	A	C	C	G	T	G	G	C	A	A	C	A	C	*E3*	G	C	A	1.44	
Dare	V		C	G	A	C	C	G	T	G	G	C	A	A	C	A	C	*E3*	G	C	A	0.81	
S-100	V		C	G	A	C	C	G	T	G	G	C	A	A	C	A	C	*E3*	G	C	A	0.51	
Ogden	VI		C	G	A	C	C	G	T	G	G	C	A	A	C	A	C	*E3*	G	C	A	0.59	
Brim	VI		C	G	A	C	C	G	T	G	G	C	A	A	C	A	C	*E3*	G	C	A	1.22	
NC-Roy	VI		C	G	A	C	C	G	T	G	G	C	A	A	C	A	C	*E3*	G	C	A	0.98	
Centennial	VI		C	G	A	C	C	G	T	G	G	C	A	A	C	A	C	*E3*	G	C	A	0.90	
Davis	VI		C	G	A	C	C	G	T	G	G	C	A	A	C	A	C	*E3*	G	C	A	0.62	
Ransom	VII		C	G	A	C	C	G	T	G	G	C	A	A	C	A	C	*E3*	G	C	A	1.52	
Roanoke	VII		C	G	A	C	C	G	T	G	G	C	A	A	C	A	C	*E3*	G	C	A	0.78	
Bragg	VII		C	G	A	C	C	G	T	G	G	C	A	A	C	A	C	*E3*	G	C	A	0.78	
Braxton	VII		C	G	A	C	C	G	T	G	G	C	A	A	C	A	C	*E3*	G	C	A	0.85	
GaSoy17	VII		C	G	A	C	C	G	T	G	G	C	A	A	C	A	C	*E3*	G	C	A	0.91	
Jackson	VII		C	G	A	C	C	G	T	G	G	C	A	A	C	A	C	*E3*	G	C	A	1.03	
NC-Raleigh	VII		C	G	A	C	C	G	T	G	G	C	A	A	C	A	C	*E3*	G	C	A	1.01	
Cook	VIII		C	G	A	C	C	G	T	G	G	C	A	A	C	A	C	*E3*	G	C	A	0.58	
