# Supplementary material for: Genetic separation of southern and northern soybean breeding programs in North America and their associated allelic variation at four maturity loci
Source: Mol Breed. 2017 Jan 11;37(1):8. doi: 10.1007/s11032-016-0611-7 (PMC5226990; doi:10.1007/s11032-016-0611-7)
Supplement: Supplementary file 6 — E1 haplotypes. Six haplotypes containing the E1 gene are presented here. The e1 mutant allele is situated in haplotype 1. E1 is not expressed in seeds, therefore sequence or expression polymorphisms among our lines are not available for direct genotyping. The E1 gene is framed in red at the proper chromosomal position in the SNP table. Note that Williams 82, which shares haplotype 1 with 27 additional landraces and milestone varieties, carries the e1-as mutant allele. The haplotypes are presented in the same format as in Suppl. Figure 1A (see legend of Suppl. Figure 1A for more information). (PDF 50 kb) [file 11032_2016_611_MOESM6_ESM.pdf]

| | | | Glyma.06G204500 | | | | Glyma.06G205000 | *E1*: Glyma.06G207800 | Glyma.06G209000 | Glyma.06G209700 | Glyma.06G210400 | | | | | | Glyma.06G211400 | | | | | | | | |
|---|---|---|---|---|---|---|---|---|---|---|---|---|---|---|---|---|---|---|---|---|---|---|---|---|---|
| Name | maturity group | haplotype | chr06: 19,275,883 | chr06: 19,277,953 | chr06: 19,278,170 | chr06: 19,278,692 | chr06: 19,372,774 | chr06: 20,207,077..20,207,940 | chr06: 20,532,951 | chr06: 20,661,062 | chr06: 20,768,647 | chr06: 20,768,767 | chr06: 20,768,938 | chr06: 20,773,898 | chr06: 20,773,958 | chr06: 20,778,554 | chr06: 21,093,956 | chr06: 21,094,533 | chr06: 21,094,699 | chr06: 21,100,565 | chr06: 21,100,574 | chr06: 21,100,644 | chr06: 21,100,762 | chr06: 21,100,867 | chr06: 21,100,890 |
| Capital | 0 | | T | G | C | A | G | *e1-as* | T | T | C | C | T | C | C | G | G | G | G | A | T | G | T | A | A |
| Mandarin (Ottawa) | 0 | | T | G | C | A | G | *e1-as* | T | T | C | C | T | C | C | G | G | G | G | A | T | G | T | A | A |
| Chippewa | I | | T | G | C | A | G | *e1-as* | T | T | C | C | T | C | C | G | G | G | G | A | T | G | T | A | A |
| Mandarin | I | | T | G | C | A | G | *e1-as* | T | T | C | C | T | C | C | G | G | G | G | A | T | G | T | A | A |
| Amcor | II | | T | G | C | A | G | *e1-as* | T | T | C | C | T | C | C | G | G | G | G | A | T | G | T | A | A |
| Amsoy | II | | T | G | C | A | G | *e1-as* | T | T | C | C | T | C | C | G | G | G | G | A | T | G | T | A | A |
| Century | II | | T | G | C | A | G | *e1-as* | T | T | C | C | T | C | C | G | G | G | G | A | T | G | T | A | A |
| Corsoy | II | | T | G | C | A | G | *e1-as* | T | T | C | C | T | C | C | G | G | G | G | A | T | G | T | A | A |
| Harcor | II | | T | G | C | A | G | *e1-as* | T | T | C | C | T | C | C | G | G | G | G | A | T | G | T | A | A |
| Harosoy | II | | T | G | C | A | G | *e1-as* | T | T | C | C | T | C | C | G | G | G | G | A | T | G | T | A | A |
| Jack | II | | T | G | C | A | G | *e1-as* | T | T | C | C | T | C | C | G | G | G | G | A | T | G | T | A | A |
| Calland | III | | T | G | C | A | G | *e1-as* | T | T | C | C | T | C | C | G | G | G | G | A | T | G | T | A | A |
| Cumberland | III | | T | G | C | A | G | *e1-as* | T | T | C | C | T | C | C | G | G | G | G | A | T | G | T | A | A |
| Ford | III | 1 | T | G | C | A | G | *e1-as* | T | T | C | C | T | C | C | G | G | G | G | A | T | G | T | A | A |
| Manchu | III | | T | G | C | A | G | *e1-as* | T | T | C | C | T | C | C | G | G | G | G | A | T | G | T | A | A |
| Oakland | III | | T | G | C | A | G | *e1-as* | T | T | C | C | T | C | C | G | G | G | G | A | T | G | T | A | A |
| Pella | III | | T | G | C | A | G | *e1-as* | T | T | C | C | T | C | C | G | G | G | G | A | T | G | T | A | A |
| Shelby | III | | T | G | C | A | G | *e1-as* | T | T | C | C | T | C | C | G | G | G | G | A | T | G | T | A | A |
| Wayne | III | | T | G | C | A | G | *e1-as* | T | T | C | C | T | C | C | G | G | G | G | A | T | G | T | A | A |
| Williams 82 | III | | T | G | C | A | G | *e1-as* | T | T | C | C | T | C | C | G | G | G | G | A | T | G | T | A | A |
| Williams | III | | T | G | C | A | G | *e1-as* | T | T | C | C | T | C | C | G | G | G | G | A | T | G | T | A | A |
| Woodworth | III | | T | G | C | A | G | *e1-as* | T | T | C | C | T | C | C | G | G | G | G | A | T | G | T | A | A |
| Zane | III | | T | G | C | A | G | *e1-as* | T | T | C | C | T | C | C | G | G | G | G | A | T | G | T | A | A |
| Bonus | IV | | T | G | C | A | G | *e1-as* | T | T | C | C | T | C | C | G | G | G | G | A | T | G | T | A | A |
| Clark | IV | | T | G | C | A | G | *e1-as* | T | T | C | C | T | C | C | G | G | G | G | A | T | G | T | A | A |
| Douglas | IV | | T | G | C | A | G | *e1-as* | T | T | C | C | T | C | C | G | G | G | G | A | T | G | T | A | A |
| Kent | IV | | T | G | C | A | G | *e1-as* | T | T | C | C | T | C | C | G | G | G | G | A | T | G | T | A | A |
| Lawrence | IV | | T | G | C | A | G | *e1-as* | T | T | C | C | T | C | C | G | G | G | G | A | T | G | T | A | A |
| Richland | II | 2 | T | G | C | C | A | *E1* | T | T | C | C | T | C | C | G | G | G | G | A | T | G | T | A | A |
| Perry | IV | | T | G | C | C | A | *E1* | T | T | C | C | T | C | C | G | G | G | G | A | T | G | T | A | A |
| Merit | 0 | | T | G | C | C | A | *E1* | C | A | C | T | T | C | T | G | G | G | G | A | T | A | T | A | A |
| Blackhawk | I | 3 | T | G | C | C | A | *E1* | C | A | C | T | T | C | T | G | G | G | G | A | T | A | T | A | A |
| Beeson | II | | T | G | C | C | A | *E1* | C | A | C | T | T | C | T | G | G | G | G | A | T | A | T | A | A |
| Mukden | II | | T | A | T | C | A | *E1* | C | A | C | T | T | C | T | G | G | G | G | A | T | A | T | A | A |
| PI 88788 | III | | T | A | T | C | A | *E1* | C | A | C | T | T | C | T | G | G | G | G | A | T | A | T | A | A |
| Adams | III | 4 | T | A | T | C | A | *E1* | C | A | C | T | T | C | T | G | G | G | G | A | T | A | T | A | A |
| Illini / A.K. (Harrow) | III | | T | A | T | C | A | *E1* | C | A | C | T | T | C | T | G | G | G | G | A | T | A | T | A | A |
| S-100 | V | | T | A | T | C | A | *E1* | C | A | C | T | T | C | T | G | G | G | G | A | T | A | T | A | A |
| FC 31745 | VI | | T | G | C | C | A | *E1* | T | T | A | C | G | T | T | A | A | A | A | T | C | G | C | G | G |
| Haberlandt | VI | 5 | T | G | C | C | A | *E1* | T | T | A | C | G | T | T | A | A | A | A | T | C | G | C | G | G |
| Tokyo | VII | | T | G | C | C | A | *E1* | T | T | A | C | G | T | T | A | A | A | A | T | C | G | C | G | G |
| Dunfield | III | | C | G | C | C | A | *E1* | T | T | A | C | G | T | T | A | A | A | A | T | C | G | C | G | G |
| PI 71506 | IV | | C | G | C | C | A | *E1* | T | T | A | C | G | T | T | A | A | A | A | T | C | G | C | G | G |
| PI 171442 | V | | C | G | C | C | A | *E1* | T | T | A | C | G | T | T | A | A | A | A | T | C | G | C | G | G |
| Dare | V | | C | G | C | C | A | *E1* | T | T | A | C | G | T | T | A | A | A | A | T | C | G | C | G | G |
| Essex | V | | C | G | C | C | A | *E1* | T | T | A | C | G | T | T | A | A | A | A | T | C | G | C | G | G |
| Hill | V | | C | G | C | C | A | *E1* | T | T | A | C | G | T | T | A | A | A | A | T | C | G | C | G | G |
| 5601T | V | | C | G | C | C | A | *E1* | T | T | A | C | G | T | T | A | A | A | A | T | C | G | C | G | G |
| Dorman | V | | C | G | C | C | A | *E1* | T | T | A | C | G | T | T | A | A | A | A | T | C | G | C | G | G |
| Hutcheson | V | | C | G | C | C | A | *E1* | T | T | A | C | G | T | T | A | A | A | A | T | C | G | C | G | G |
| Ogden | VI | | C | G | C | C | A | *E1* | T | T | A | C | G | T | T | A | A | A | A | T | C | G | C | G | G |
| Brim | VI | | C | G | C | C | A | *E1* | T | T | A | C | G | T | T | A | A | A | A | T | C | G | C | G | G |
| Davis | VI | | C | G | C | C | A | *E1* | T | T | A | C | G | T | T | A | A | A | A | T | C | G | C | G | G |
| Dillon | VI | | C | G | C | C | A | *E1* | T | T | A | C | G | T | T | A | A | A | A | T | C | G | C | G | G |
| Hood | VI | | C | G | C | C | A | *E1* | T | T | A | C | G | T | T | A | A | A | A | T | C | G | C | G | G |
| Lee | VI | | C | G | C | C | A | *E1* | T | T | A | C | G | T | T | A | A | A | A | T | C | G | C | G | G |
| NC-Roy | VI | | C | G | C | C | A | *E1* | T | T | A | C | G | T | T | A | A | A | A | T | C | G | C | G | G |
| Tracy | VI | 6 | C | G | C | C | A | *E1* | T | T | A | C | G | T | T | A | A | A | A | T | C | G | C | G | G |
| Young | VI | | C | G | C | C | A | *E1* | T | T | A | C | G | T | T | A | A | A | A | T | C | G | C | G | G |
| Arksoy | VI | | C | G | C | C | A | *E1* | T | T | A | C | G | T | T | A | A | A | A | T | C | G | C | G | G |
| Ralsoy | VI | | C | G | C | C | A | *E1* | T | T | A | C | G | T | T | A | A | A | A | T | C | G | C | G | G |
| Centennial | VI | | C | G | C | C | A | *E1* | T | T | A | C | G | T | T | A | A | A | A | T | C | G | C | G | G |
| Pickett | VI | | C | G | C | C | A | *E1* | T | T | A | C | G | T | T | A | A | A | A | T | C | G | C | G | G |
| CNS | VII | | C | G | C | C | A | *E1* | T | T | A | C | G | T | T | A | A | A | A | T | C | G | C | G | G |
| Roanoke | VII | | C | G | C | C | A | *E1* | T | T | A | C | G | T | T | A | A | A | A | T | C | G | C | G | G |
| Volstate | VII | | C | G | C | C | A | *E1* | T | T | A | C | G | T | T | A | A | A | A | T | C | G | C | G | G |
| Bragg | VII | | C | G | C | C | A | *E1* | T | T | A | C | G | T | T | A | A | A | A | T | C | G | C | G | G |
| Braxton | VII | | C | G | C | C | A | *E1* | T | T | A | C | G | T | T | A | A | A | A | T | C | G | C | G | G |
| GaSoy17 | VII | | C | G | C | C | A | *E1* | T | T | A | C | G | T | T | A | A | A | A | T | C | G | C | G | G |
| Hagood | VII | | C | G | C | C | A | *E1* | T | T | A | C | G | T | T | A | A | A | A | T | C | G | C | G | G |
| Jackson | VII | | C | G | C | C | A | *E1* | T | T | A | C | G | T | T | A | A | A | A | T | C | G | C | G | G |
| NC-Raleigh | VII | | C | G | C | C | A | *E1* | T | T | A | C | G | T | T | A | A | A | A | T | C | G | C | G | G |
| Ransom | VII | | C | G | C | C | A | *E1* | T | T | A | C | G | T | T | A | A | A | A | T | C | G | C | G | G |
| Cook | VIII | | C | G | C | C | A | *E1* | T | T | A | C | G | T | T | A | A | A | A | T | C | G | C | G | G |
